# Frequency and Risk Factors of Endocrine Complications in Turkish Children and Adolescents with Sickle Cell Anemia

**DOI:** 10.4274/tjh.2012.0001

**Published:** 2013-03-05

**Authors:** Samim Özen, Selma Ünal, Neslihan Erçetin, Bahar Taşdelen

**Affiliations:** 1 Mersin Maternity and Children’s Hospital, Department of Pediatric Endocrinology, Mersin, Turkey; 2 Mersin University Medical School, Department of Pediatric Hematology, Mersin, Turkey; 3 Mersin Maternity and Children’s Hospital, Biochemistry Laboratory, Mersin, Turkey; 4 Mersin University, Department of Biostatistics, Mersin, Turkey

**Keywords:** Sickle cell disease, Nutritional status, Endocrine system diseases, children

## Abstract

**Objective:** To define frequency and risk factors of abnormalities in growth, puberty, thyroid function, and bone and carbohydrate metabolisms in children and adolescents with sickle cell disease (SCD).

**Materials and Methods:** Endocrine problems including short stature, puberty and thyroid disorders, and carbohydrate and bone metabolisms in 50 Turkish children and adolescents with SCD were evaluated. Relationships among sex, disease type, blood transfusions, exchange and exacerbation frequency, ferritin levels, and endocrine pathologies were investigated.

**Results:** The mean age of the study group was 13.1±2.9 years. Weights and heights of 12 participants (24%) were below -2 standard deviations and 4 participants (8%) had malnutrition. Mean difference (±standard deviation) between bone and chronological age of patients was -1.73±1.86 years. Fifty percent of patients had at least one endocrine abnormality other than vitamin D deficiency and insufficiency. Hypergonadotropic hypogonadism in 3 patients (6%), hypogonadotropic hypogonadism in 1 female patient (2%), and small testicular volume in respect to age in 3 male patients (8.5%) were seen. Growth hormone deficiency was detected in 1 (2%) female patient, and hypothyroidism was diagnosed in 3 patients (6%; 1 central case, 2 cases of primary hypothyroidism). At vertebral level, 5 patients (11.1%) had osteopenia and 1 patient (2.2%) had osteoporosis, while 5 patients (11.1%) had osteopenia at femur neck level. The most common endocrine abnormality was vitamin D deficiency. 25-Hydroxyvitamin D was deficient in 63.2% and insufficient in 18.4% of patients. Sex, disease type, blood transfusion frequency, exacerbation frequency, and ferritin levels were not related to endocrine pathologies. As the age was increased, standard deviation scores of femur neck bone mineral density was decreased (r =-0.56; p<0.05). Vitamin D was lower in patients whose weights and/or heights were below -2 standard deviations from the mean (p<0.05).

**Conclusion:** Endocrine organ dysfunctions are commonly detected in children and adolescents with SCD, and vitamin D deficiency is the most commonly encountered endocrine disorder. Regular follow- ups of patients for endocrine complications, starting from early ages of patients, and initiation of appropriate treatments will elongate expectancy and quality of life.

**Conflict of interest:**None declared.

## Abstract (tr)

**Amaç:** Orak hücre anemili (OHA)’li çocuk ve ergenlerde büyüme, puberte, tiroid, kemik ve karbonhidrat metabolizmazı bozuklularının sıklığı ve risk faktörlerinin belirlenmesi amaçlanmıştır.

**Yöntemler:** 50 OHA’li Türk çocuk ve adolesanda vücut ölçümleri yanında boy, puberte, tiroid, karbonhidrat ve kemik metabolizması bozuklarını içeren endokrin sorunlar değerlendirildi. Cinsiyet, hastalık tipi, kan transfüzyonu, exchange ve atak sıklığı, ferritin düzeyi ile endokrin patolojiler arasında ilişki araştırıldı.

**Bulgular:** Yaş ortalaması 13,1±2,9 yıl olan hastaların 12’sinde (%24) ağırlık ve boyu -2 standart deviasyon (SD) altında ve 4’ünde (%8) malnütrisyon bulundu. Hastaların ortalama (±SD) kemik yaşı-takvim yaşı farkı -1,73±1,86 yıl idi. Vitamin D eksikliği dışında hastaların %50’sinde en az bir endokrin bozuklukluk vardı. 3 hastada (%6) hipergonadotropik hipogonadizm, 1 kız hastada (%2) hipogonadotropik hipogonadizm, 3 erkek hastada (%8,5) ise yaşa göre testis volum küçüklüğü saptandı. 1 kız hastada (%2) büyüme hormonu eksikliği, 3 hastada (%6) hipotiroidi (1 santral, 2 primer hipotiroidi) tanısı konuldu. Vertebral düzeyde hastaların 5’inde (%11,1) osteopeni, 1’inde (%2,2) osteoporoz, femur boynu düzeyinde ise 5’inde (%11,1) osteopeni saptandı. Hastalarda en sık görülen endorin bozukluk vitamin D eksikliği idi. 25- Hidroksivitamin D %63,2 hastada eksik, %18,4 hastada yetersiz bulundu. Cinsiyet, hastalık tipi, kan transfüzyonu sıklığı, atak sıklığı, ferritin düzeyi ile endokrin patolojiler arasında ilişki saptanmaz iken, yaş arttıkça femur boynu kemik mineral yoğunluğu SDS azaldığı (r=-0,56; p<0,05), ağırlık ve/veya boyu -2 SD altında olanlarda D vitamini değerinin daha düşük olduğu saptandı (p<0,05).

**Sonuç:** OHA’li çocuk ve ergenlerde endokrin organ bozuklukları sık görülür ve en sık endokrin sorun D vitamini eksikliğidir. Hastaların erken yaşlardan itibaren endokrin komplikasyonlar açısından düzenli olarak takip edilmesi ve uygun tedavilerin verilmesi yaşam süreleri ve yaşam kalitelerini artıracaktır.

## INTRODUCTION

Sickle cell disease (SCD) is a hemolytic anemia, characterized by abnormal hemoglobin production of autosomal recessive inheritance. SCD may lead to acute and chronic tissue ischemia and many organ dysfunctions due to intermittent small vascular obstructions [1,2]. While studies related to malnutrition, growth retardation, and pubertal development retardation were more frequently reported in pediatric patients with SCD [2,3], studies in gonadal insufficiency, thyroidal disorders, and bone metabolism were conducted with the adulthood and endocrine organ dysfunctions more frequently reported in SCD patients, especially in studies performed at adulthood [1,2,3]. The physiopathology of metabolic and endocrine disorders in these patients is not clear yet. Investigators propose that endocrine organ dysfunctions in SCD patients may be caused by iron overload due to recurrent blood transfusions or disruptions of tissue vitalization during vaso-occlusive crisis and inflammatory mediators [1,2,3]. On the other hand, as observed in other chronic diseases, malnutrition, which may negatively affect growth and development in childhood, is commonly encountered in SCD [4]. The present study aimed to determine frequency and risk factors of abnormalities in growth, development, puberty, thyroid health, and bone and carbohydrate metabolism in children and adolescents with SCD.

## MATERIALS AND METHODS

This study included 50 Turkish children and adolescents with SCD who were below 18 years of age and applied regularly to the Pediatric Hematology Department of the Medical School of Mersin University and the Mersin Maternity and Children’s Hospital Department of Pediatric Endocrinology Outpatient Clinic between December 2009 and October 2011. Disease type, annual vaso-occlusive and sickle crises, blood transfusion and exchange frequencies, and serum ferritin levels were obtained from the medical records; patients with fewer than 5 vaso-occlusive crises and/or sickle crises a year were defined as having “fewer attacks”, and those with 5 or more were defined as having “frequent attacks”. Patients with fewer than 5 annual blood transfusions were defined as “less transfusion required”, and those with 5 and above were defined as “frequent transfusion required”. Endocrinological evaluations of patients were performed by the same pediatric endocrinology specialist. Height was measured using a rigid stadiometer. Weight was measured unclothed to the nearest 0.1 kg using a calibrated balance scale. Body mass index (BMI) was calculated by the formula of weight (kg) divided by height squared (m2), and standard deviation scores (SDSs) of weight, height, and BMI were also calculated for Turkish children [5]. Short stature was defined as a height SDS value below -2 standard deviations (SDs) and malnutrition was defined as a BMI SDS level below -2 SDs [5]. The pubertal status of each subject was determined using the Tanner criteria [6]. All testicular volumes were measured with a Prader orchidometer and compared with normal values [7].Three-day estimated diet records and vitamin D and calcium intake of subjects were computed. 

**Endocrinological Evaluation and Stimulation Test**

Morning fasting blood samples were obtained for serum insulin, glucose, calcium (Ca), phosphorus (P), alkaline phosphatase (ALP), 25-hydroxyvitamin D (25(OH)D), and intact parathyroid hormone (iPTH) analysis. Subjects were then asked to drink a solution containing 1.75 g/kg (maximum: 75 g) of glucose, and blood samples were obtained again for 120-min glucose and insulin measurements. Serum glucose, Ca, P, and ALP levels were measured using a Beckman Coulter UniCel^®^ automatic analyzer. Ca, P, and ALP levels were defined by the normal ranges of the kits used. A fasting blood glucose level of ≥100 mg/dL was considered impaired fasting glucose and a fasting blood glucose level of ≥126 mg/dL (7 mmol/L) was considered diabetes mellitus. In the oral glucose tolerance test, a 120-min glucose level of 140-199 mg/dL was considered impaired glucose tolerance and a 120-min glucose level of ≥200 mg/dL (11.1 mmol/L) was considered diabetes mellitus [8].

Insulin, free triiodothyronine (fT3), free thyroxine (fT4), thyroid stimulating hormone (TSH), luteinizing hormone (LH), follicle stimulating hormone (FSH), estradiol (E2), testosterone (T), growth hormone (GH), 25(OH)D, and iPTH measurements were performed by electrochemiluminescence method using the Roche^®^ Diagnostic (Germany) Modular E-170 device and Cobas kits. 

Insulin resistance (IR) using the homeostasis model assessment (HOMA) model was calculated as fasting insulin (µU/mL) × fasting glucose (mmol/L)/22.5. Insulin resistance was defined by HOMA-IR values of ≥3.16 in pubertal subjects and HOMA-IR of ≥2.67 in prepubertal subjects [9,10].

The corresponding normal values for the 2.5 and 97.5 percentiles of fT3, fT4, and TSH were 2.0-4.4 pg/mL, 0.93-1.7 ng/dL, and 0.27-4.2 µIU/mL, respectively. TSH insufficiency was diagnosed by fT4 of less than 0.8 ng/dL in combination with a normal or below normal TSH level and thyrotropin-releasing hormone (7 µg/kg) test. Normal values for LH, FSH, E2, and T were evaluated according to pubertal stages. Hypogonadotropic hypogonadism was suspected when both LH and FSH responses to LH-RH stimulation (100 µg/m^2^) were flat in patients of pubertal age. Basal and stimulated levels of TSH, prolactin (PRL), FSH, and LH were determined at sequential time points (15, 30, 60, and 90 min after stimulation). Pharmacological GH stimulation was tested by insulin tolerance test and levodopa. A peak value below 10 ng/mL in 2 tests was regarded as confirmatory for the diagnosis of GH deficiency [11]. 

Serum 25(OH)D levels of ≥75 nmol/L (30 ng/mL) were defined as normal, 50-75 nmol/L (20-30 ng/mL) as insufficient, and <50 nmol/L (20 ng/mL) as deficient [10]. The normal range for iPTH levels was 10-65 pg/mL according to the kit’s manual, and therefore iPTH levels above 65 pg/mL were considered as indicative of hyperparathyroidism. In order to avoid any seasonal differences, all measurements of 25(OH)D and iPTH were performed in the summer. Serum ferritin levels of 500 ng/mL and above were defined as “high”.

Bone age was determined by same pediatric endocrinologist using a left-wrist radiograph and was assessed according to the method of Greulich and Pyle [13]. The difference between bone age and calendar age (ΔBACA) was defined by subtracting the chronological age from the bone age at the time of wrist radiography. The bone mineral density (BMD; g/cm^2^) of the lumbar spine and femoral neck was measured with dual energy X-ray absorptiometry (Hologic QDR 4500A Fan Beam X-ray Bone Densitometer, Hologic, Bedford, MA, USA). Z scores of measured values were calculated according to the standards of Turkish children developed by Goksen et al. [14]. Patients with Z scores according to age, sex, and height between -1 and -2 were accepted as osteopenic and those at and below -2 were accepted as osteoporotic [15]. 

The authors confirmed in writing that they had complied with the World Medical Association Declaration of Helsinki regarding ethical conduct of research involving human subjects and/or animals. The study was approved by the local ethics board and informed consent was obtained from the families of all patients.

**Statistics**

Study data were analyzed using SPSS for Windows 15.0 (SPSS Inc., Chicago, IL, USA). Frequency tables for categorical variables and descriptive statistics for numerical variables were applied. Given the cross-tabulation statistics between groups, level of significance was detected by chi-square test. In numerical comparisons, the t-test was used in normally distributed variables, whereas the Mann-Whitney U test was used for abnormally distributed variables. The Spearman correlation coefficient was calculated to test abnormally distributed variables. Statistical significance was accepted as p<0.05.

## RESULTS

A total of 50 patients, who had mean age of 13.1±2.9 years (range: 4.3-17.8 years) and comprised 15 females (30%) and 35 males (70%), were enrolled in the study. Five patients were younger than 10 years of age. Descriptive information of the patients is presented in Table 1.

While 30 of the patients (60%) had HbSS anemia, 20 of them had the Hb S-beta type. Attack frequencies were “fewer attacks” in 29 patients (58%) and “frequent attacks” in 21 patients (42%), whereas blood transfusions were required “less” in 37 patients (74%) and “more frequently” in 13 patients (26%). While 16 patients (32%) had exchange requirements, 15 patients (30%) received regular chelating treatment. With a mean serum ferritin level of 510.9±817 g/mL, the serum ferritin level of 15 patients (30%) was 500 ng/mL or above. Serum ferritin level was higher than 1000 ng/mL only in 4 patients. There were no cardiac or liver disorders in patients due to iron accumulation. 

SDS values of weight, height, and BMI of cases were -1.02±1.04, -1.03±1.38, and -0.41±1.14, respectively. The mean difference (±SD) between bone and chronological age (ΔBACA) of patients was -1.73±1.86 years. In 12 (24%) patients (4 females, 8 males), weight and height values were below -2 SDS. Among those patients, 7 were prepubertal (stage 1) and 5 were pubertal (Tanner stage of 2 or greater), and the mean age was 11.5±3.5 years old. ΔBACA SDS was -1.92±1.01 years in these patients. Four patients (8%) had malnutrition. 

The endocrine complication frequency of the patients was 50% (25 complications in 50 patients), with at least 1 endocrine organ dysfunction in 18 patients, 2 different complications in 2 patients, and 3 different complications in 1 patient. Detected pubertal abnormalities were hypergonadotropic hypogonadism in 3 patients (2 females and 1 male; 6%) and hypogonadotropic hypogonadism in 1 female patient (2%). The 3 hypergonadotropic patients were 15.2 (female), 14.5 (female), and 16.5 (male) years old with no breast development in the girls. The male patient had no secondary sexual characteristics and both testicular volumes were 2 mL. Basal LH levels were 51.2 and 63.4 mIU/mL (normal: 0.1-12 mIU/mL) and E2 levels were below 12 pg/mL (normal: 25-345 pg/mL) in the girls. In the male patient with hypergonadotropic hypogonadism, basal LH and testosterone levels were 75.1 mIU/mL (normal: 02.-5 mIU/mL) and 4.2 ng/dL (normal: 100-320 ng/dL), respectively. A girl of 15 years and 8 months with hypogonadotropic hypogonadism had no breast development or menarche. In this patient, peak LH level was detected as 0.4 mIU/mL, the basal E2 level was 13.1 pg/mL, and other pituitary hormone levels and pituitary magnetic resonance imaging (MRI) were normal. Three (8.5%) male patients also had small testes. These patients were 8, 10.5, and 11.5 years old. In these patients, both testicular volumes were below 2 mL and peak LH values were detected as 2.0, 2.1, and 1.6 mIU/mL, respectively. 

GH deficiency was detected in 1 (2%) female patient (13 years old, Tanner stage 2, bone age of 8 years and 10 months). Her weight and height SDSs were -1.3 and -3.2, respectively. In this patient, serum insulin-like growth factor-1 (IGF-1) level was 35 ng/dL (below -2 SD), peak GH values were 2.4 and 1.5 ng/mL in 2 different GH stimulation tests, and pituitary MRI was normal. 

Hypothyroidism was detected in 3 patients (1 central and 2 primary hypothyroidism). Central hypothyroidism was detected in a 15.9-year-old male. His weight, height, and pubertal status (Tanner stage 5) were normal. He had low fT4 (0.72 ng/dL) and normal TSH (1.0 µIU/mL) levels. In this patient, TRH test showed TSH insufficiency (basal and peak TSH levels: 1.3 µIU/mL and 4.55 µIU/mL, respectively). 

In the evaluation of 45 patients whose BMD values were measured, L1-L4 BMD Z score was 0.47±1.33 SD and femur neck BMD Z score was 0.54±1.16 SD. At the vertebral level, 5 patients (11.1%) had osteopenia and 1 had (2.2%) osteoporosis, whereas at the femoral level, osteopenia was detected in 5 patients (11.1%) (Table 2). Average daily vitamin D and calcium intakes in all patients were calculated as 232.12±110.21 IU and 685.34±324.12 mg, respectively. Average daily vitamin D and calcium intakes of patients with osteopenia and/or osteoporosis, which were calculated as 168.11±91.12 IU and 456.32±198.76 mg, respectively, were statistically significantly low (P=0.032 and P = 0.043, respectively). Ca, P, and ALP values were within normal limits in all cases. Vitamin D and iPTH levels were assessed in 38 patients. The detected mean 25(OH)D level (19.31±9.68 ng/mL) was very low. For 25(OH)D, 63.1% (n=24) of patients had deficiency and 18.4% (n = 7) had insufficiency (Table 2). The mean iPTH level was 44.4±17.4 pg/mL in the study group. However, in 18.4% (7/38) of patients, the parathormone level was high (mean iPTH level: 76.8±10.1 pg/mL). Mean iPTH level in vitamin D deficient and insufficient subjects was higher compared to those with normal vitamin D status (47.01±12.91 vs. 36.24±11.25, p=0.026). 

No diabetes was detected in patients, but fasting insulin resistance was detected in 3 patients (6%). There was no correlation among sex, disease type, blood transfusion, exchange and attack frequency, ferritin level, and endocrine pathologies. However, femur neck BMD SDS decreased as age increased (r =-0.56, p<0.05), and vitamin D values were lower in patients who had weight and/or height below -2 SD (p<0.05). 

## DISCUSSION

Endocrine dysfunction in hematological diseases like thalassemia and SCD, which progresses with hemolysis and iron storage, can be encountered commonly and at early ages. The physiopathology of endocrine organ pathology is not still clear in patients with SCD. However, iron storage due to recurrent and frequent transfusions, or ischemia due to vaso-occlusive crises and inflammatory mediators during ischemia, are proposed as reasons for endocrine dysfunction [1,2,3]. In the present study, growth, puberty, thyroid problems, and bone and carbohydrate metabolism disorders were evaluated.

Growth retardation is the most commonly encountered endocrine disorder in patients with SCD. In previous studies, height, weight, and BMI values were demonstrated to be prominently lower in patients with SCD when compared to healthy controls [16,17]. Growth retardation in these patients may be related to factors like nutritional disorders [18], chronic inflammatory process, hypermetabolism in bone marrow [19,20], and hypogonadism [2,3]. In recent studies, it was found that abnormalities in GH-insulin-like growth factor-1 and IGF-1 binding protein 3 axis may cause growth retardation in patients with SCD, in whom growth is normal at birth but starts to retard after 1-2 years of age [21,22]. In a study conducted of 102 cases, heights of 54% of sickle cell anemia patients were reported to be lower than -2 SD [23]. In another study, growth retardation was reported in 24% of 76 patients with SCD [24]. When body measurements were evaluated according to Turkish children’s standards in our study, weight (-1.02±1.04) and height (-1.03±1.38) SDS values were below the mean standards. Weight and height values were below -2 SD in 12 patients (24%), and 4 patients (8%) had malnutrition. Bone ages in the study group were prominently retarded (ΔBACA SDS: -1.73±1.86 years). In a 13-year-old female patient, severe short stature (height SDS: -4.3), low IGF-1 level (35 ng/dL, below -2 SD), retarded bone age (8 years and 10 months), and GH deficiency were detected. 

In 4 patients (8%) in our study group, hypogonadism was confirmed by LH-RH test along with clinical appearance. Etiology of hypogonadism in patients with SCD is not clear yet. However, reasons like primary testicular/ovarian insufficiency, hypothalamic and/or pituitary dysfunction, zinc deficiency, and constitutional pubertal retardation may be responsible [2,3,25]. Three (2 females and 1 males) out of our 4 hypogonadal patients had hypergonadotropic hypogonadism, and 1 female patient had hypogonadotropic hypogonadism. Three male patients of prepubertal age also had small testes. Both testicular volumes were below 2 mL in these patients. We have been following these patients in our pediatric endocrine outpatient clinic for pubertal status.

Hypothyroidism was detected in 3 patients (6%; 2 patients had primary and 1 patient had central) in our study group. The etiology of thyroid dysfunction in SCD is not clear; however, most affected patients have received multiple transfusions consistent with severe iron overload. Autopsy reports in some patients have shown significant iron deposition in the thyroid gland, suggesting that the etiology of the primary thyroid failure might well be transfusional hemosiderosis and subsequent cellular damage to the thyroid gland [2]. The reports of thyroid assessment in patients with SCD have been inconsistent. Abnormal thyroid function studies have been reported in patients with SCD [26,27]. Stimulation with TSH-releasing hormone showed increases in TSH that were significantly greater in SCD compared with controls and thus were suggestive of primary thyroid failure [28].

Out of 38 patients whose 25(OH)D levels were measured, 24 (63.1%) had vitamin D deficiency, 7 (18.4%) had vitamin D insufficiency, and iPTH was high in 18.4% (n=7/38). Serum iPTH levels of subjects in the vitamin D deficient and insufficient groups were statistically significantly higher than those of subjects in the vitamin D sufficient group. This might be an important finding, as a high iPTH level is an indirect indicator of vitamin D deficiency. Otherwise, low calcium intake can influence serum iPTH levels even in patients with vitamin D sufficiency [12]. In our study, there was no correlation between iPTH levels and daily calcium intake. Further large-scale studies are required to draw the final conclusion on this topic. There are increasing data demonstrating low serum levels of vitamin D among HbSS children, possibly linked to decreased dietary intake and in some cases to seasonal variability in food intake [29]. Buison et al. [29] and Rovner et al. [30] reported low levels of serum 25(OH)D in children (65% and 90%, respectively) with sickle cell anemia HbSS compared with their age-matched and racially matched peers. In our study, vitamin D deficiency in children and adolescents with SCD was detected at rates higher than that of the general Turkish population (43.8%) as reported by Hatun et al. [31]. Daily vitamin D intake was insufficient for patients in our study (232.12±110.21 IU). Vitamin D deficiency in children and adolescents with SCD can result from causes like sedentary lifestyle due to chronic disease, chronic inflammation, and hypermetabolic status along with malnutrition and insufficient intake [2,3,32]. Among 44 patients who were evaluated for BMD, 10 patients (22.7%) had osteopenia and 1 (2.2%) had osteoporosis. Low BMD has been reported in male and female children and adults with SCD. In a study of 32 adults with SCD (mean age of 34 years), 72% had low BMD at 1 or more anatomic sites and 40% were classified as osteoporotic [33]. Markers of bone formation are elevated whereas bone resorption is decreased in children with SCD compared with healthy children [34]. Additional mechanisms and risk factors for osteopenia in SCD include delayed puberty and low accrual of peak bone mass, bone microinfarcts resulting from repeated sickle crises, chronic illness with immobilization, and calcium, vitamin D, and other nutritional deficiencies [35,36]. In our study, average daily vitamin D and calcium intakes of patients with osteopenia and/or osteoporosis were statistically significantly lower than those without BMD disorders (p=0.032 and p=0.043, respectively). There was no relationship among crisis frequency, chelating treatment, transfusion anamnesis, and osteopenia/osteoporosis. Considering these results, we conclude that vitamin D intake disorder is most likely responsible for osteopenia/osteoporosis development in our patient population.

Although none of the patients who were evaluated by oral glucose tolerance test had diabetes or impaired glucose tolerance, fasting insulin resistance was detected in 3 patients (6%). A report from the Multi-Center Study of Iron Overload, a 5-year prospective study in Canada, the United States, and the United Kingdom, reported that diabetes mellitus affects 2% of patients with SCD. In logistic regression analysis, the strongest predictor of diabetes was the length of time since patients had started receiving transfusions. After adjusting for diagnosis (e.g., SCD and thalassemia) and serum ferritin levels in the model, both duration and age at which the subject began chronic transfusion remained significant. The analysis revealed that for every 10 years of transfusion use, transfused subjects with SCD had 2.5 times greater odds of diabetes [27]. In our study, BMI values of the 3 patients with insulin resistance were above 85%. None of the patients with normal weight had insulin resistance. We did not observe any insulin resistance or diabetes, because our study was conducted during childhood. 

In conclusion, endocrine organ dysfunctions are commonly encountered in children and adolescents with SCD, and vitamin D deficiency is the most commonly encountered endocrine disorder. Regular follow-ups of patients for endocrine complications starting at early ages and initiation of appropriate treatments will elongate expectancy and quality of life. 

**Conflict of Interest Statement**

The authors of this paper have no conflicts of interest, including specific financial interests, relationships, and/ or affiliations relevant to the subject matter or materials included.

## Figures and Tables

**Table 1:**
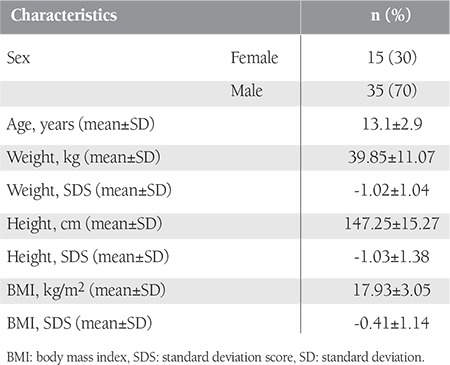
Descriptive information of the study group.

**Table 2:**
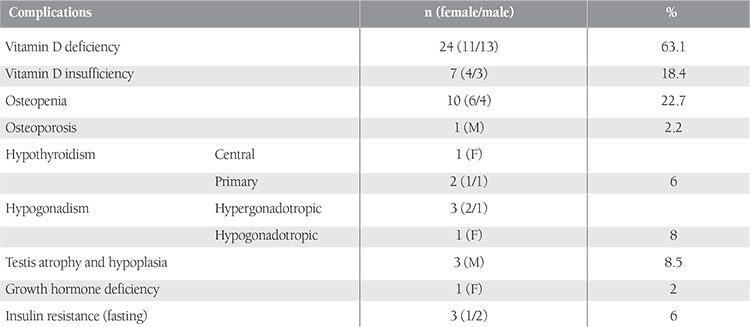
Distribution of endocrine problems and complications in patients with SCD.
